# Green Energy Harvester from Vibrations Based on Bacterial Cellulose

**DOI:** 10.3390/s20010136

**Published:** 2019-12-24

**Authors:** Carlo Trigona, Salvatore Graziani, Giovanna Di Pasquale, Antonino Pollicino, Rossella Nisi, Antonio Licciulli

**Affiliations:** 1 Department of Electrical, Electronics and Computer Engineering (DIEEI), University of Catania, Viale Andrea Doria 6, 95125 Catania, Italy; 2 Dipartimento di Scienze Chimiche, Dipartimento di Scienze Chimiche and INSTM UdR, University of Catania, Viale Andrea Doria 6, 95125 Catania, Italy; giovanna.dipasquale@dii.unict.it; 3 Department of Civil Engineering and Architecture (DICAR) and INSTM UdR, University of Catania, Viale Andrea Doria 6, 95125 Catania, Italy; apollicino@unict.it; 4 BioFaber srl, via Luigi di Savoia 19, 72023 Mesagne (BR), Italy; rossella.nisi@unisalento.it; 5 Dipartimento di Ingegneria dell’Innovazione, University of Lecce, Via per Arnesano, 73100 Lecce, Italy; antonio.licciulli@unisalento.it

**Keywords:** green energy harvesting, bacterial cellulose, mechanical vibrations, biodegradability, ionic liquids, conducting polymers, bio-factory

## Abstract

A bio-derived power harvester from mechanical vibrations is here proposed. The harvester aims at using greener fabrication technologies and reducing the dependence from carbon-based fossil energy sources. The proposed harvester consists mainly of biodegradable matters. It is based on bacterial cellulose, produced by some kind of bacteria, in a sort of bio-factory. The cellulose is further impregnated with ionic liquids and covered with conducting polymers. Due to the mechanoelectrical transduction properties of the composite, an electrical signal is produced at the electrodes, when a mechanical deformation is imposed. Experimental results show that the proposed system is capable of delivering electrical energy on a resistive load. Applications can be envisaged on autonomous or quasi-autonomous electronics, such as wireless sensor networks, distributed measurement systems, wearable, and flexible electronics. The production technology allows for fabricating the harvester with low power consumption, negligible amounts of raw materials, no rare elements, and no pollutant emissions.

## 1. Introduction

The availability of low-cost electronics will enable the diffusion of smart systems, which will invade virtually any aspect of people’ s every-day life, with relevant changes in production processes and lifestyle [1]. Electronic smart systems will both further diffuse in traditional application fields (such as the process industry or the automotive applications) and conquer new ones (e.g., agriculture or smart homes) [1,2]. Due to this diffusion process in unconventional environments, new functionalities will be required for electronics. One of the commonly stated requirements deals with systems power autonomy, which will characterize all those applications where power lines are not available or are inconvenient [3]. In such, conditions systems need to be capable of scavenging energy from the environment [4,5]. 

Different strategies have been already proposed, that are capable of collecting energy from ambient sources, such as solar power [6], thermic gradients [7], acoustic [8], RF [9], and mechanical vibrations and motions [10].

Though energy harvesting from motion sources poses challenges because of the frequency range of the available mechanical vibration and of the level of energy that can be collected, it is among the most widely investigated ones, because of the large availability of motion sources that could be usefully exploited. Specific solutions have been proposed for collecting energy from mechanical vibrations, with suitable strategies, mechanisms, and converters [11,12,13].

Along with the needing for new functionalities, the pervasive diffusion of low-cost electronics will pose environmental challenges. Sustainable production strategies will be required, both in terms of used raw materials and energy and produced pollutants. The after-life of electronics will be, also, a relevant issue. Since low-cost electronics will become available, devices will be quickly disposed of. Landfilling and even pollution phenomena will result if e-waste will not be properly processed.

Polymeric electronics can represent a suitable technology, capable of giving meaningful answers to the needing of energy scavenging, as well as of “greener electronics”. More specifically, Electro Active Polymers (EAPs), a class of polymer-based composites, which are capable of mechanoelectrical conversion have been already proposed for energy scavenging from mechanical vibrations [14,15]. Tough being based on polymers, the systems previously mentioned, are not based on “green” materials [16]. 

Recently the interest of the scientific community has been focused on new classes of materials that better fit the needing of greener economics. Cellulose is among the most promising compounds since it is a cheap and green bio-derived polymer, with exceptional mechanical and electrical properties. Bacterial Cellulose (BC), a kind of cellulose produced by specific classes of bacteria, can represent an even better solution. It is, in fact, characterized by environmentally respecting production processes [17]. Due to such promising properties, both the electromechanical [18] and mechanoelectrical transduction properties of BC have been recently investigated [19,20,21]. Taking a cue from the mechanoelectrical transduction capabilities of BC-based compounds [19,20,21], this paper investigates the possibility of scavenging energy from low-frequency mechanical vibrations.

A BC-based composite, with Ionic Liquids (ILs) and polymeric electrodes, has been investigated to realize a green, eco-friendly, and mostly biodegradable vibration energy harvester. More specifically, the composite is mounted in a cantilever configuration and generates an electrical signal, when vibrating. Energy across a resistive load is finally produced.

The investigation has been performed at the mechanical resonance frequency of the beam, i.e., 17 Hz. A base acceleration of about 9 m/s^2^ ≈ 1 g has been applied. The corresponding measured maximum power, delivered on an optimal resistive load, was 80 pW. Though technologies exist that are capable of delivering a larger power, for similar working conditions, the aim of the paper is demonstrating the possibility of fabricating power harvesters by using a green production process.

The proposed harvester could be used in those applications where line powering is impossible or not convenient so that alternative powering sources are needed. Nevertheless, to the best of the authors’ knowledge, no technology is yet available, at the considered geometrical scale, capable of allowing fully autonomous systems. Harvesters proposed so far, work as sustainers that charge accumulation systems for discontinuously working electronics. An example of this class of applications is represented by wireless sensor networks, using sensing nodes that do not need continuously working. In such cases, the sensor is turned on when enough energy has been collected to perform the measuring operations and to transmit the acquired data. In this scenario, the proposed harvester has the added value of being almost totally biodegradable, so that its disposal is not the main concern, even if a huge number of harvesters is disseminated in large areas. 

The paper is organized as follows: Section 2 describes materials, the prototype, and the methods used to characterize the composite. Section 3 reports and discusses the obtained results, while the discussions and conclusions are given in Section 4.

## 2. Materials and Methods

### 2.1. The BC Description and Properties

In the last years, BC has attracted increasing attention as a material for sensors realization, due to its higher purity compared to plant cell cellulose and to its crystalline structure.

BC is a nanostructured biopolymer produced by the fermentation of Gluconoacetobacter species, which can form, through self-assembly in the bacteria culture process, an interconnecting porous network of nanofibers. A pure and crystalline cellulose nanofibril tissue can be obtained from these bacterial colonies, which thus form “miniaturized biofabric” with high energy and matter efficiency and high regenerative potential.

BC is structured in a web-like, 3D network. It consists of ribbon-shaped nanofibers with a typical diameter in the range from 10 to 50 nm.

It is a potential reinforcing agent for the realization of environmentally-friendly electronics, due to its high degree of polymerization (14,400 Da), crystallinity (89%), specific area (37 m^2^/g), excellent moldability, and high mechanical properties [22,23]. 

Furthermore, BC hydrophilic nature (99% of BC is water), flexibility, non-toxicity, good biocompatibility, and wide availability have promoted its use in different fields from food and paper industry, to biomedical applications, and green electronics.

The presence of surface hydroxyl groups in BC makes this material suitable for functionalization with various nanomaterials, further expanding potential application fields.

### 2.2. The BC Preparation

BC used in the reported activity was produced by Biofaber srl (Italy) [22]. It was produced, in a kind of bio-industry, from static bacterial cell culture, with different sources of carbon and nitrogen, obtained by agro-alimentary waste fermentation [22], in the form of hydrogel pellicles. More specifically, BC was produced by a symbiosis of bacteria (Gluconoacetobacter) and yeasts (Zygosaccharomyces), which assemble a cellulose hydrogel in just a few weeks.

In order to obtain BC, commercial black tea bags and sucrose, a symbiosis of bacteria and yeast strain were used. Sodium hydroxide (NaOH) and acetic acid were purchased from Sigma-Aldrich and used without further modification. 

Pure BC pellicles were obtained from the fermentation process of the sweetened black tea in the presence of bacteria and yeasts. The bacterial culture is composed of an upper cellulosic pellicle and a lower liquid broth, as described in [22]. 

The culture medium was prepared by adding 70 g of sucrose and 4 g of black tea to boiling water (1 L). Then, the mixture was left to steep for 15 min and, after removing the tea, the pH value of the broth was adjusted to 2.7–3.0, by adding 10 mL of acetic acid for each liter of the broth. The addition of acetic acid at the beginning of the fermentation process prevents the formation of molds and protects against undesirable microorganisms. Finally, the Gluconoacetbacter and the Zygosaccharomyces strain (1 mL/L) were added to the cooled tea broth. The fermentation process was carried out at room temperature (28 °C) for 15 d in a static culture condition. In this period, pellicles of cellulose were grown on the surface of the broth. 

**Figure 1:**
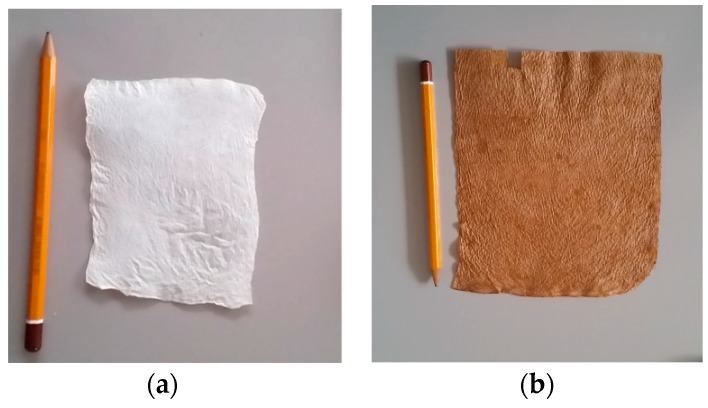
Pictures of different aspects of Bacterial Cellulose (BC). (**a**) BC gel after purification. (**b**) BC dried on gypsum at environmental temperature, without any purification.

The pellicles of nanofibrillated BC were thoroughly washed with distilled water and boiled in 0.5 M NaOH solution for 2 h, in order to extract the endotoxin from the BC samples. Then, pellicles were placed in a depyrogenated sample container and washed with endotoxin-free water four times. The BC pellicles were finally deposited on a flat gypsum plate and dried in an oven, for eliminating any residual water content. Samples of the BC were, finally, cut to the required size. Pictures showing changes in BC appearance produced by the processing are reported in Figure 1. More specifically, Figure 1a shows a dried BC, after the purification step with NaOH. Figure 1b shows, for the matter of comparison, a BC sheet dried on gypsum, which has not undergone the purification phase. Both samples, shown in Figure 1, have been dried at room temperature. 

**Figure 2:**
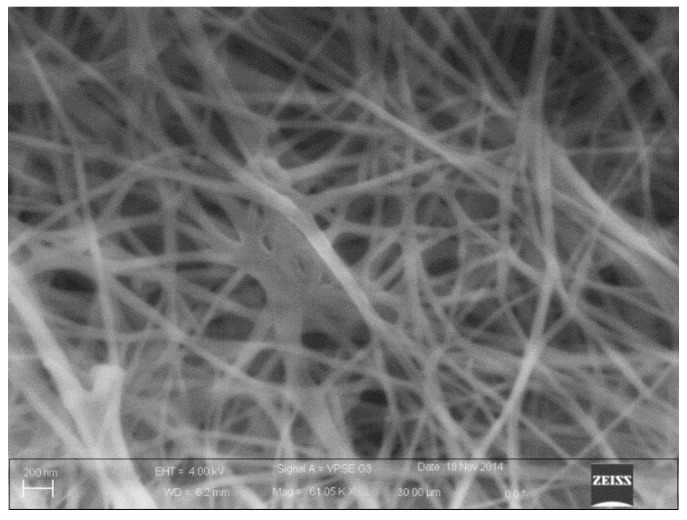
Scanning electron microscopic morphology of pure BC.

Morphological characterizations of the samples were performed on a Zeiss (Sigma VP; Carl Zeiss, Oberkochen, Germany) FESEM. To perform the analyses, a thin slice was cut from the BC with a sharp stainless steel blade and placed on the carbon tape of the sample holder. A SEM image of the produced BC is shown in Figure 2.

### 2.3. The Prototype Used as Energy Harvester from Mechanical Vibrations

**Figure 3:**
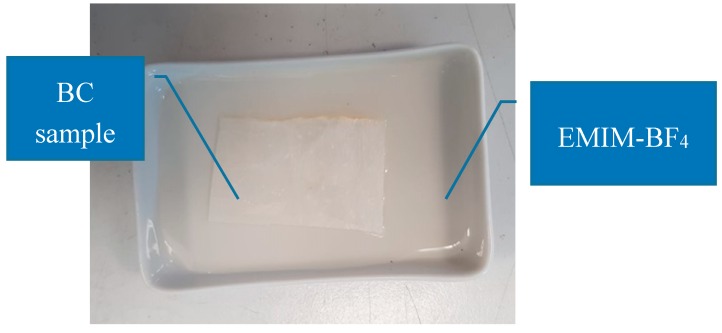
A picture of the BC sample, during the impregnation phase.

Samples (5 × 5 cm) were cut from larger BC sheets. Thermogravimetric measurements in static air at a heating rate of 20 °C/min, carried out on samples cut from the same large BC sheets, showed that at 120 °C the sample weight loss was on average equal to about 4%. Since at this temperature no cellulose degradation occurs, the weight loss was attributed to absorbed water and then, before IL impregnation, the samples were further dried in an oven overnight at 65 °C to eliminate any water reabsorbed during the storage. The dried membranes were soaked for 24 h with 1-Ethyl-3-methylimidazolium tetrafluoroborate (EMIM-BF_4_) in an alumina rectangular tray put in a desiccator containing CaCl_2_. EMIM-BF_4_, an IL whose melting temperature is 15 °C, was purchased by Sigma-Aldrich and used without any further modification. It is a salt where 1-Ethyl-3-methylimidazolium (EMIM) is the cation ionically bonded to tetrafluoroborate (BF_4_) as counterion. The impregnated sample was then dried in a vacuum oven for 24 h at a temperature of 65 °C. A picture showing the IL impregnation of the purified and dried BC is shown in Figure 3. 

**Figure 4:**
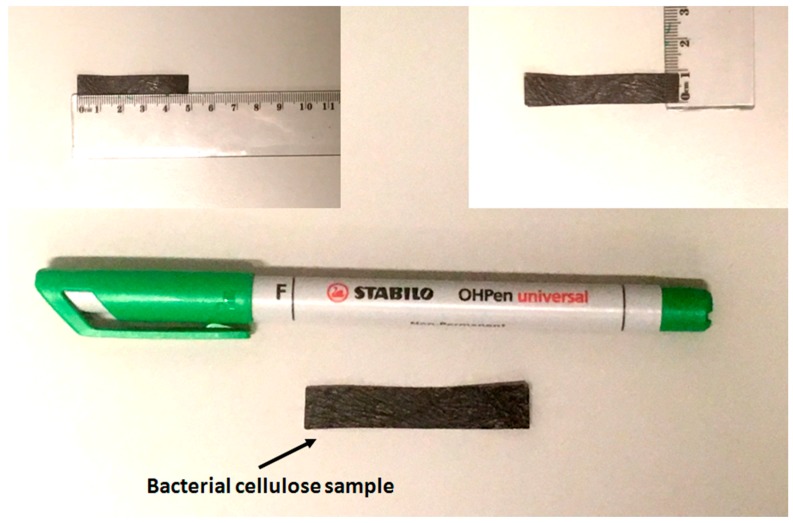
Picture of the BC-based device used as a green energy harvester.

Then, BC/EMIMBF4/PEDOT devices were fabricated by depositing four layers (24 μm) of poly (3,4-ethylenedioxythiophene) polystyrene sulfonate (PEDOT-PSS) water dispersion on both sides of the BC, by using a film spreader. After each deposition step, the membranes were dried in an oven for 5 min. In order to eliminate short circuits between the PEDOT-PSS electrodes, the membranes were trimmed to a rectangular shape. Figure 4 shows the transducer used as the green energy harvester from mechanical vibrations. The characteristics of the conceived device are summarized in Table 1.

## 3. Experimental Results and Discussion

An experimental setup has been realized by measure for the characterization of the green energy harvester. It is widely reported in the literature that this class of composites (i.e., ionic-polymer composites) reacts to mechanical flexural deformation. More specifically, the cantilever configuration is generally adopted for power harvesting [14]. In such a configuration, the base of the beam is forced by vibrating motion and a beam deflection is obtained.

- An electrodynamic shaker (TIRA TV 50009), for mechanically exciting the structure.
- A signal generators HP33120A, for impressing a suitable waveform to the shaker.
- An oscilloscope, LeCroyWaveRunner 6050, for acquiring the signals.
- A single-axis accelerometer, used to monitor the imposed vibrations.
- Two laser sensors, used for the measurement of the sensor displacement.

**Figure 5:**
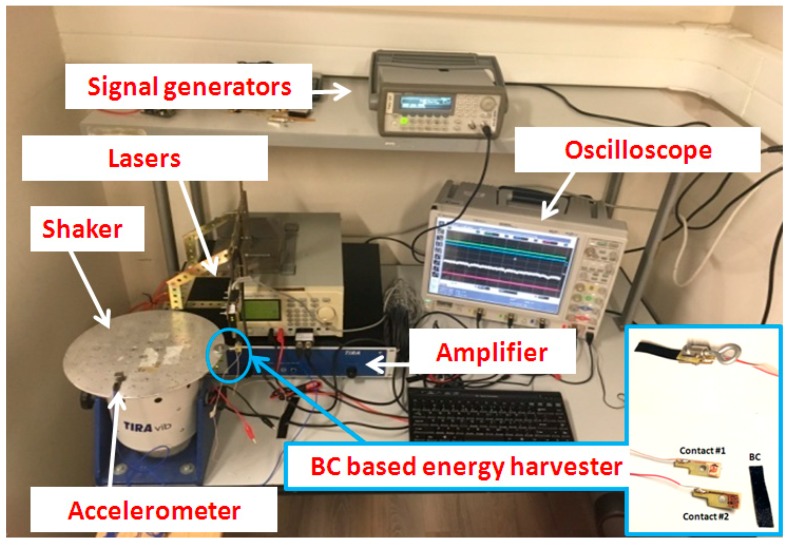
Experimental setup used for studying and characterizing the BC-based vibration energy harvester.

Resistors of different values have been used as the load for characterizing the BC-based energy harvester in terms of maximum power transfer. Figure 5 shows a picture of the entire setup with, in the inset, a detail of the BC-based harvester, its contacts, and the support, used to fix it in the shaker plate.

When a linear harvester configuration is implemented, the best performance, in terms of collected power is obtained when the beam is forced to vibrate at its mechanical resonance frequency [10,24]. Moreover, in a previous investigation, some of the authors demonstrated that when the BC-IL composite is forced by a constant amplitude input signal, a maximum output signal is produced when the system is forced at its mechanical resonant frequency [20]. According to such considerations, the investigation of the harvester has been performed at the beam mechanical resonant frequency. This required a preliminary investigation by using the experimental setup. 

**Figure 6:**
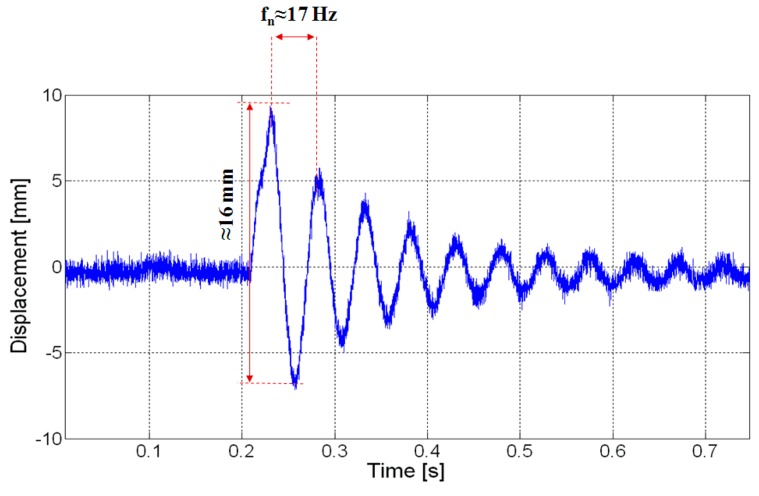
Displacement evaluated at the tip of the beam in the presence of a mechanical step impressed through the shaker.

**Figure 7:**
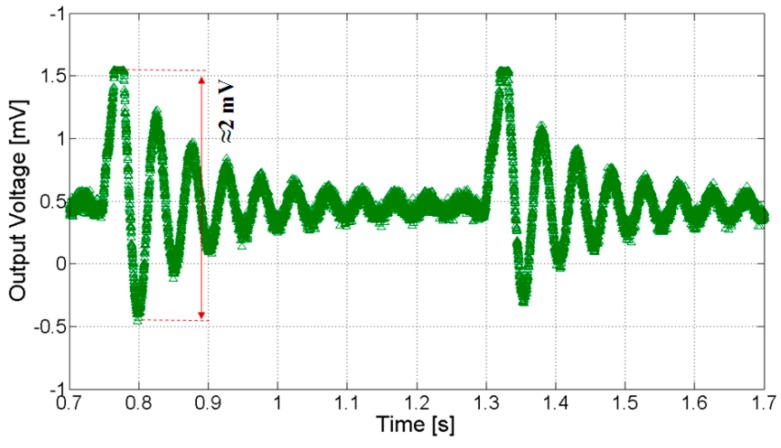
The output voltage of the BC in the presence of a mechanical step.

Figure 6 reports a recording of the tip displacement of the BC, as measured by one of the two laser sensors. The graph shows a displacement of about 16 mm when a mechanical step is applied. Furthermore, a damped frequency of about 17 Hz has been estimated. Figure 7 reports the corresponding output voltage produced by the transducer, in open circuit conditions. It is possible observing that a maximum output voltage of about 2 mV can be obtained in correspondence of the aforementioned tip displacement.

**Figure 8:**
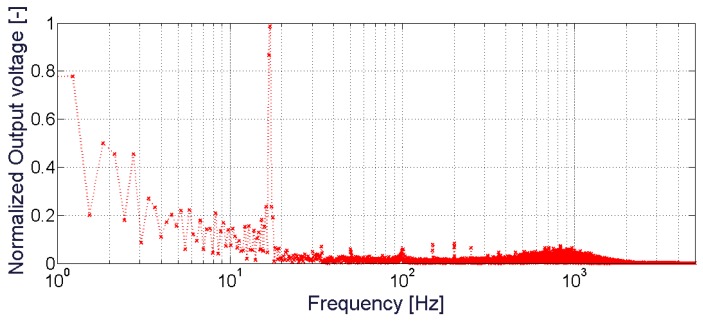
Fast Fourier Transform (FFT) of the BC output voltage in the presence of sinusoidal mechanical vibration, at 17 Hz. Data have been normalized to the maximum value of the spectrum.

Figure 8 shows the magnitude of the FFT of the BC output signal, estimated in the presence of a sinusoidal mechanical vibration, at about 17 Hz and level of acceleration of ~9 m/s^2^. The graph shows two main contributions; one at the resonant frequency of the device (~17 Hz) and a DC signal, mainly given by the intrinsic properties of the BC. It represents a static contribution produced because of the capacitive nature of the BC composite. It is worth noting that this effect has been already observed during the characterization of the BC as a sensor [21].

**Figure 9:**
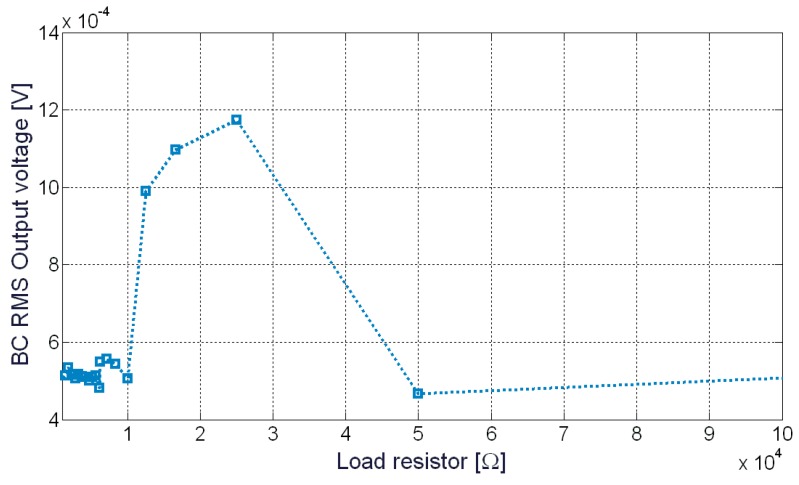
BC output voltage as a function of several resistive loads. The system has been excited with a sinusoidal waveform at 17 Hz and an Root Mean Square (RMS) acceleration level of about 9 m/s^2^.

In order to characterize the device as an energy harvester, resistive loads, with different values, have been used. The optimal load for the vibration energy harvester has been, therefore, searched for. In this context, Figure 9 shows the output voltage of the BC-based energy harvester, in the presence of a sinusoidal waveform having a frequency of about 17 Hz and a vibration level of about 9 m/s^2^. This value of frequency has been selected in order to find the optimal load. It must be observed that this value of frequency is a consequence of the displacement analysis (see Figure 6), where a damped frequency of about 17 Hz has been observed.

As can be noted, a maximum value of voltage can be observed around 25 kΩ. In this specific case, the RMS voltage corresponds to about 1.2 mV.

**Figure 10:**
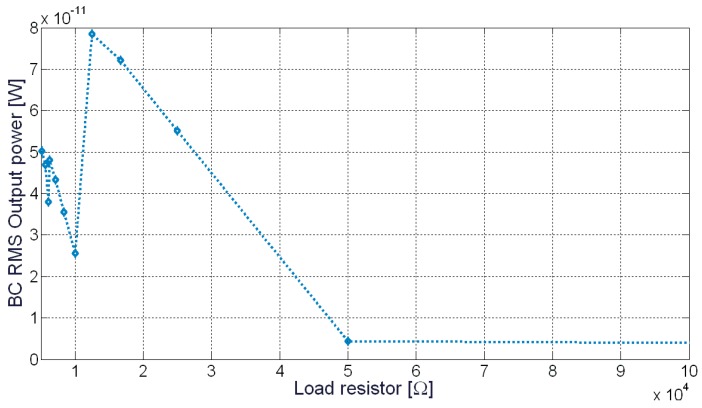
The BC output power as a function the resistive load value.

Figure 10 shows the maximum power transfer analysis, correlated with the load resistor. As it can be observed, the value corresponds to about 12 kΩ and a maximum power of about 80 pW can be generated.

In order to study the performance of the green energy harvester here proposed, the efficiency of the transducer has been also estimated in accordance with the metrics used for vibration energy scavengers [24].

**Figure 11:**
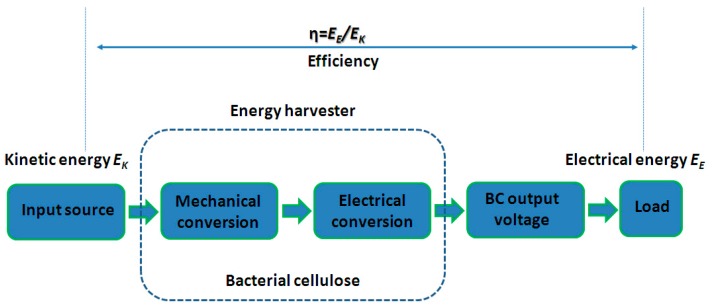
Block diagram of the green energy harvester architecture.

Figure 11 shows the block diagram of the entire harvesting process, starting from the mechanical source, through the BC-based transducer, to the load. More specifically, the input kinetic energy (*E_K_*) is a sinusoidal mechanical signal. It represents the input of the green energy harvester, which is composed of two main parts, the mechanical conversion as a function of the imposed external vibrations, and an electrical conversion based on the transduction properties of the BC. As a consequence, an output voltage will be generated and power will be dissipated across the load. The power dissipated at the load is produced by an electrical energy *E_E_*.

The efficiency is expressed as [24]:

(1) $$\eta =\frac{E_{E}}{E_{K}}=\frac{\frac{V_{BC}^{2}}{R}\;t}{m\;a\;x}$$

where *V_BC_* is the generated voltage through the BC in the presence of a sinusoidal mechanical vibration at 17 Hz (mechanical resonant frequency of the green oscillator), *R* is the load resistor, and its value corresponds to the optimal load value estimated during the characterization described above. By using these two parameters, the power can be estimated. By multiplying the power value by the observation time (*t*) the electrical energy (*E_E_*) is obtained.

In presence of a sinusoidal vibration having an acceleration level of about 9 m/s^2^ and frequency of 17 Hz, considering the displacement (*x*), evaluated thanks to the laser sensor, the acceleration level (*a*), measured by the accelerometer, and considering the mass of the harvester (*m*), the efficiency corresponds to about 10^−4^%.

Table 2 summarizes the performance of the green energy harvester. A comparison of the performance of the proposed BC-IL-based harvester, with competing technologies, is moreover reported in Table 3. The table shows both the order of magnitude of the power that can be collected by different technologies and specific values reported in the literature are given. It is worth noticing, anyway, that indicated values greatly depend on the specific working conditions described in the indicated references. As can be observed, the power that can be obtained by using the proposed device is smaller with respect to other technologies, including PZT, silicon-based solutions, MEMS, and piezo-ionic transducers. Nevertheless, this characteristic is counterbalanced by the environmental impact of the proposed harvester. The proposed BC-IL-based harvester is low-cost, produced by a green fabrications process, which greatly reduced the corresponding CO_2_ emissions. Moreover, it is mostly biodegradable and eco-friendly.

As a final remark, techniques exist that allow for improving the power harvested level. More specifically, it has been reported in the literature that by using nonlinear configurations, an increase up to six times with respect to corresponding linear configurations can be obtained [29]. The authors are working at developing such nonlinear BC-based power harvesters.

## 4. Conclusions

In this paper, the possibility of harvesting energy by using a BC-IL-based composite is demonstrated. More specifically, the composite is fabricated by a biodegradable base material and by using a green production process. The system is, then, mounted in a cantilever configuration and its energy harvesting performance from mechanical vibrations is studied by an experimental setup. 

The power collected by using the proposed BC-IL-based system is lower with respect to competing technologies. Nevertheless, the implemented technology has relevant advantages in terms of its environmental impact. The device is, in fact, fabricated by using cellulose, produced by bacteria, without any consumption of nonrenewable raw materials. Moreover, the production process does not imply relevant energy consumption, nor pollutants release. The proposed harvester is, then, realized by absorbing commercially available ILs, which are considered green solvents. Finally, in the present form, conducting polymers are used for the realization of the electrodes, which lack biodegradability. Nevertheless, studies have been reported in the literature showing the possibility of realizing biodegradable conducting polymers. The use of such new conducting polymers in the future realizations of the BC-IL-based harvesters can further increase the relevance of the proposed technology as a contribution to the realization of the next generation greener electronics.

## Figures and Tables

**Table 1:** Characteristics of the BC transducer used as a green energy harvester.

Parameter	Value	Unit of Measurement
Length	49	mm
Width	9	mm
Thickness	0.3	mm
f_n_	17	Hz ^1^
m	0.035	g ^1^
k	0.4	N/m ^2^
Maximum Displacement	16	mm

^1^ measured, ^2^ estimated through analytical model.

**Table 2:** Performance of the green energy harvester.

Parameter	Value	Unit of Measurement
Generated power	80	pW
Density	0.13	cm^3^
Generated Voltage	1.2	mV
Operative frequency	17	Hz
Efficiency	10^−4^	%

**Table 3:** Comparison of performance for several energy harvesters based on different technologies.

Motion Transducer	Power Level (Reported Values Are Indicative, and Grealy Depend on the Specific Configuration)	Environmental Friendliness	Ref.
Lead zirconate titanate (PZT)	Hundreds of microwatts. e.g., 0.9 mW are reported in [25].	Low	[25]
Silicon-based (MEMS)	Power up to tens of picowatts can be obtained. 3.6 pW are reported, e.g., in [26].	Medium	[26]
Lead-free PZT	Few microwatts can be obtained. 1.6 µW are reported in [27].	Medium	[27]
Polyvinylidene fluoride (PVDF)	In the range from nanowatts to microwatts. In [28], 93.6 µW have been measured	Low	[28]
Ionic polymer‑metal composite IPMC	From the order of picowatts to the order of nanowatts. e.g., 1 and 3 nW were envisaged in [14,15], respectively;	Medium	[14,15]
BC	Up to tens of picowatts.	High	This work
